# Editorial: Advances in the social determinants of health for college students with ADHD

**DOI:** 10.3389/fpsyt.2026.1830924

**Published:** 2026-08-11

**Authors:** Sarah O’Neill, Anthony L. Rostain, Khushmand Rajendran, Anne-Claude V. Bedard

**Affiliations:** 1 Department of Psychology, City College of New York (CUNY), New York, NY, United States; 2 Department of Psychiatry, Cooper Medical School of Rowan University, Camden, NJ, United States; 3 Department of Psychiatry and Behavioral Health, Cooper University Health Care, Camden, NJ, United States; 4 Department of Social Science & Human Services, Borough of Manhattan Community College (CUNY), New York, NY, United States; 5 Ontario Institute for Studies in Education, The University of Toronto, Toronto, ON, Canada

**Keywords:** ADHD, college, neurodivergence, post-secondary, social determinants of health

## Adhd among postsecondary students

Postsecondary education confers economic advantages to individuals and wider communities (1) and is an opportunity for personal growth and identity development (2). Globally, over 260 million students are enrolled in higher education (3), an increasing proportion of whom have ADHD (4, 5).

Core responsibilities of college students include managing their daily habits, self-care, lifestyle, and academic duties. For those with ADHD, developmental and contextual factors, including emotion regulation and/or executive functioning difficulties, reduced support from family and educators, and high levels of both perceived *and* lived experiences of discrimination, amplify the challenges of transitioning to college (6–8).

## The importance of a global perspective

Outcomes for college students with ADHD are further influenced by social determinants, including socioeconomic status, access to assessments and treatments or appropriate academic support, the quality of relationships, and stigma (9–12). However, current literature may not generalize to students in low- and middle-income countries (LMICs), low-resource communities, or culturally distinct educational contexts. This special issue begins to address this gap by reporting findings from Africa [Ethiopia (13) and South Africa (14)], the Middle East [Israel (15)], and the South Pacific [New Zealand (16)].

## Key findings from this special issue

Across all studies, compared to students with no/low ADHD, students with elevated ADHD behaviors had academic difficulties. Students reported greater anxiety, less knowledge, and less favorable attitudes toward statistics (15); lower academic self-efficacy (16); fewer study hours for their exams, lower GPAs, and greater overall test anxiety (13). Students also shared that their attention span was shorter than the typical lecture duration (14). These findings align with previous studies indicating disparate academic outcomes for college students with ADHD relative to their non-ADHD peers (17), including lower grades and a greater likelihood of dropping out (9).

Feeling constantly behind or as if one is always “putting out fires” is highly stressful (18) and negatively impacts self-concept (19). Turner and Harty (16) demonstrated that New Zealand students with ADHD had lower self-esteem ratings across all daily assessment intervals than their non-ADHD peers. This was associated with more difficulty initiating tasks, reported by participants as procrastination. Not surprisingly, ADHD was also associated with more severe depression and anxiety. South African students gave voice to their lived experiences of high anxiety in academic and social situations, which led them to use maladaptive coping strategies, such as self-isolation, masking, and substance use (14).

Behavioral and emotional dysregulation interacted with and compounded each other. For example, South African students reported that anxiety exacerbated their concentration problems (14). New Zealand students with ADHD spent less time with friends than their non-ADHD peers, despite time spent with friends being associated with higher self-esteem (16).

Notably, ADHD presentation was affected by social determinants. In Ethiopia, students from rural communities were 4.49 times more likely to screen positive for ADHD than their peers from urban centers. Compared to students whose mothers had attended college, those students whose mothers could not read/write were more than twice as likely to screen positive for ADHD; and students with a history of childhood infection were significantly more likely to screen positive for ADHD than those without (13).

### Methodological considerations

The majority of the articles in this special issue relied on screening questionnaires or self-reported ADHD diagnoses rather than comprehensive clinical evaluations. However, not all individuals who score high on a screening measure will meet the diagnostic criteria for ADHD. Furthermore, screening questionnaires rarely consider childhood behaviors, leaving uncertainty as to whether the childhood-onset criterion for ADHD is met. Finally, the sensitivity and specificity of a single measure are generally too low to be adequate for clinical decision-making (20). At the same time, young adults’ narratives illuminate the difficulties often faced in obtaining an ADHD evaluation, including high cost, long waitlists, referral challenges, and a lack of connection with healthcare providers (21). In addition, stigma surrounding ADHD may limit young people’s engagement with service providers.

In all the studies, the participants were attending a four-year college/university, but community college students with ADHD also struggle (22) and may face additional stressors (23) that amplify the impact of ADHD on functioning. To our knowledge, there are no studies on the prevalence of ADHD or the experiences of students with ADHD attending vocational training schools; this is an area for future research.

## New research directions

Shifting from an impairment-based framework to a strength-based one is recommended. For example, New Zealand students with ADHD spent more time at university than students without ADHD, which was associated with higher self-esteem (16). Scholars have proposed ways in which tertiary institutions can develop the necessary infrastructure to support neurodivergent students (24, 25). In addition to expanding mental health and educational assistance through wellness centers or disability services (25), supports could be systematically integrated into courses (e.g., instructions in multiple formats, chunking of assignments) (24), likely benefiting all students, but particularly those with ADHD. Ryder et al. (15) showed that despite having higher test anxiety and lower self-efficacy, students with ADHD performed similarly to their non-ADHD peers in statistics. It is essential to communicate this to students with ADHD, given the relations between self-efficacy and academic performance and internalizing psychopathology (26). Similarly, instructors may benefit from learning how ADHD impacts students’ academic functioning. Students with ADHD perceive their relationships with faculty to be worse than students without ADHD do, which in turn is related to lower self-efficacy and student engagement (27). Faculty efforts to improve their understanding of students’ experiences and to adopt neuroinclusive pedagogical practices (28) may go some way toward mitigating these outcomes.

Hearing directly from students themselves is essential. Mpetha et al. (14) presented powerful narratives of students’ lived experiences, and in doing so, identified potential treatment targets. Considering these students’ experiences, shame and internalized beliefs of self-doubt and defectiveness are likely important to address in a therapeutic context (29). Group treatment may be particularly beneficial in showing students that they are not alone in their experiences (30).

Inclusive research practices, including establishment of community advisory boards and co-design of research studies by neurodivergent postsecondary students, are also essential (31). Community-based participatory research built on a foundation of mutual respect, equality, and collaboration offers real potential for a greater understanding of ADHD, its related challenges, *and* individuals’ strengths (32).

Finally, postsecondary students with ADHD may have comorbid diagnoses or cognitive profiles that impact their academic outcomes, use of coping strategies, and treatment response. In particular, research on autism and twice-exceptional status is lacking, and these are important areas for future research.

## Educational and treatment implications

Campuses must establish better resources for students with ADHD. Low-cost, accessible assessments are an essential first step. Tertiary education providers need to develop infrastructure to screen and evaluate college students for ADHD. Many students have no history of clinical evaluation, especially those from historically marginalized, disinvested, or resource-constrained communities (30, 33). However, institutions commonly require formal documentation of a diagnosis for students to access services. One of the South African participants stated that the lack of a diagnostic evaluation (due to unaffordable cost) made it hard to explain their behavior to peers (14). Notably, girls/women and people of color are underdiagnosed, misdiagnosed, or receive their diagnoses later than White boys/men, which is linked to poorer outcomes (34, 35). It is important that educators and clinicians be attuned to these issues and to how current diagnostic tools may contribute to these disparities, to help ensure these differences in health outcomes are not perpetuated.

Accommodations (36) and psychotherapeutic interventions should be culturally tailored (37), adapted for individuals with ADHD (38–40), and developed with input from students (41). In addition, neurodivergent-affirming approaches may result in greater student engagement and more effective outcomes.

ADHD remains a highly stigmatized neurotype (42, 43). Kassaw et al. (13) hypothesized that stigma interacts with ADHD behaviors to exacerbate impairment. Individuals may mask their behaviors (44), refrain from seeking healthcare (45), or choose not to disclose their diagnosis (21, 45). Students may be reluctant to inform their instructors and/or peers or seek out accommodations due to fear of negative evaluation or differential treatment (46). Psychoeducation and destigmatization efforts are needed in campus communities. In addition, parents play a role in helping their children transition to college while fostering their autonomy (47). Parents (and, in turn, the parent-adult child relationship) (11) may benefit from psychoeducation that ADHD is a neurodevelopmental disorder and that ADHD behaviors do not represent character flaws.

## Conclusions

This special issue presents four contributions from around the world that highlight commonalities and local nuances in ADHD presentation and impairment among postsecondary students. It calls for researchers not only to investigate group-level differences, but also to consider contextual and cultural factors that modulate outcomes and represent avenues for novel and effective support.
